# Flow Synthesis of Nature-Inspired Mitochondria-Targeted Phenolic Derivatives as Potential Neuroprotective Agents

**DOI:** 10.3390/antiox11112160

**Published:** 2022-10-31

**Authors:** Desirée Pecora, Francesca Annunziata, Sergio Pegurri, Pasquale Picone, Andrea Pinto, Domenico Nuzzo, Lucia Tamborini

**Affiliations:** 1 Department of Pharmaceutical Sciences, University of Milan, Via Mangiagalli 25, 20133 Milan, Italy; 2 Istituto per la Ricerca e l’Innovazione Biomedica (IRIB), CNR, Via Ugo La Malfa 153, 90146 Palermo, Italy; 3 Department of Food, Environmental and Nutritional Sciences, University of Milan, Via Celoria 2, 20133 Milan, Italy

**Keywords:** mitochondria, neuronal cells, natural phenolic acids, triphenylphosphonium cation, flow chemistry, Cyrene

## Abstract

A series of phenolic derivatives designed to selectively target mitochondria were synthesized under flow conditions starting from natural phenolic acids. The two-step continuous flow protocol, performed in Cyrene, a bioavailable dipolar aprotic solvent, allowed the isolation of the MITO compounds in moderate to good yields. The MITO compounds obtained, as a first step, were tested for their safety by cell viability analysis. The cytocompatible dose, in human neuronal cell line SH-SH5Y, depends on the type of compound and the non-toxic dose is between 3.5 and 125 µM. Among the seven MITO compounds synthesized, two of them have shown interesting performances, being able to protect mitochondria from oxidative insult.

## 1. Introduction

**Figure 1:**
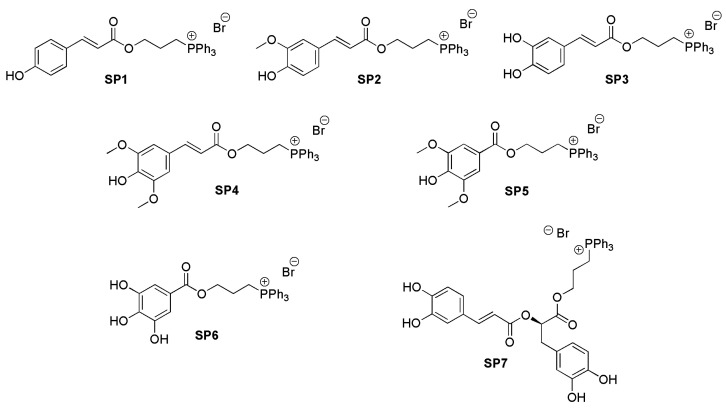
Structures of the synthesized MITO-phenolic derivatives **SP1-7**. **SP1**: MITO-coumaric ester; **SP2**: MITO-ferulic ester; **SP3**: MITO-caffeic ester; **SP4**: MITO-sinapic ester; **SP5**: MITO-syringic ester; **SP6**: MITO-gallic ester; **SP7**: MITO-rosmarinic ester.

Mitochondria are important intracellular organelles that are present in eukaryotic cells and play a key role in energy production and regulation of cell-stress pathways. Mitochondrial defects or dysfunctions are associated with the onset of several neurological and cardiovascular diseases [1,2,3,4,5,6]. In fact, in pathological conditions, impaired mitochondrial electron transport chain activity increases reactive oxygen species (ROS) production, leading to mitochondrial-driven injuries. Mitochondrial oxidative stress contributes to cell damage such as lipid peroxidation, protein oxidation, and DNA lesions. Because of the negative membrane potential of the mitochondrial inner membrane, positively charged compounds accumulate in the mitochondrial matrix against their concentration gradient [7]. Various lipophilic cations, including alkyl triphenylphosphonium cations, rhodamine, cyanine cations, and cationic peptides, can be attached to the bioactive compound of interest to improve its mitochondrial uptake. The highly negative inner membrane potential of mitochondria represents a driving force for the attraction of cationic molecules and appears an efficient strategy to effectively target mitochondria with antioxidants, nitric oxide-liberating molecules, and probes. Such a negative membrane potential is not found in any other subcellular compartments and thus constitutes a very selective way to deliver compounds to these organelles. However, it is generally considered that the uptake of cations by mitochondria is not only caused by their positive charge but also by their hydrophobicity [8]. Among all systems and organs, the central nervous system (CNS) is particularly exposed to oxidative stress because of its high oxygen consumption (20% of the total oxygen), weak antioxidant system, and the large amount of ATP produced [9]. Consequently, oxidative stress is considered to underlie many neurodegenerative diseases. Indeed, Aβ peptide, a characteristic marker of Alzheimer’s disease (AD), interacts with different cellular structures such as mitochondria. Growing evidence suggests a link between mitochondrial dysfunction and AD [10]. Therefore, the development of compounds capable of targeting mitochondria and restoring their function is highly appealing and needed [11]. In particular, mitochondria-targeted antioxidants can inhibit cellular death, preventing aging and the development of chronic diseases. In this context, we developed a continuous flow synthesis for the obtainment of a series of phenolic derivatives to selectively target mitochondria (Figure 1, compounds **SP1-7**). Natural phenolic acids, i.e., coumaric acid, sinapic acid, syringic acid, ferulic acid, gallic acid, caffeic acid, and rosmarinic acid, were selected for their interesting biological properties, including their antioxidant activity and neuroprotective effects [12,13,14]. As lipophilic moiety for mitochondrial delivery, we linked a triphenylphosphonium cation (TPP+) to the phenolic acid through the formation of an ester. For this study, as linker, a propyl chain was selected, as successfully exploited in different MITO-derivatives, such as MITO-curcumin [15], MITO-doxorubicin [16], and MITO-chlorambucil [17]. TPP^+^-based mitochondrial targeting shows several advantages compared to other approaches, including the stability of the TPP+ moiety in biological systems, a combination of lipophilic and hydrophilic properties, and low chemical reactivity towards cellular components, as demonstrated by mitochondria-targeted quinone (MITOQ), which has been shown to be relatively safe in humans, thereby enhancing the potential clinical significance of this class of molecules [18,19]. However, the chemical esterification of phenolic acids or alcohols is difficult due to heat sensitivity and susceptibility to oxidation in alkaline media of phenolic compounds. Moreover, chemical synthesis is usually unselective, resulting in unwanted side reactions and formation of byproducts, and consequently requires several protection/deprotection steps of phenolic groups, leading to poor atom economy and generating extra waste to dispose of [20]. Therefore, we designed a two-step continuous flow protocol in Cyrene, a biobased dipolar aprotic solvent [21,22], to obtain the desired MITO compounds. Cyrene has been successfully used for many important chemical reactions, such as amidation reactions, synthesis of ureas, SN_2_ and carbon–carbon couplings, and its use under flow conditions could significantly expand its applicability [23]. As a first step, the cytocompatibility of compounds **SP1-7** was evaluated after dose–effect stimulation and the IC_50_ was calculated after 24 h. Then, the effect of the compounds on mitochondria perturbation induced by treatment of neuronal SH-SY5Y human cells with an oxidant agent (*tert*-butyl hydroperoxide; TBH) was investigated. In particular, the ability to protect cellular mitochondria from oxidative insult was analyzed by MTS assay. This method is based on the conversion of MTS into formazan, which depends on mitochondrial respiration/activity and indirectly serves to assess the cellular energy capacity that is related to the number of viable cells.

## 2. Materials and Methods

Reagents and solvents were obtained from commercial suppliers and were used without further purification. Rosmarinic acid was extracted following a previously reported procedure [24]. NMR spectra were recorded on a Varian Gemini 300 MHz spectrometer using the residual signal of the deuterated solvent as internal standard. ^1^H chemical shifts (*δ*) are expressed in ppm and coupling constants (*J*) in hertz (Hz). Continuous flow biotransformations were performed using a Series E Vapourtec flow reactor equipped with reactor coils (10 mL). The temperature sensor sits on the wall of the reactors. Pressure was controlled by using back-pressure regulators. HPLC analyses were performed using a Waters 1525 Binary HPLC Pump, equipped with a Waters 2489 UV-vis detector (Waters, Milford, MA, USA) and a C18 column (100 Å, 3.5 µm, 4.6 mm × 75 mm); flow rate 1 mL/min; λ = 254 nm. Mobile phase: (A) H_2_O + 0.1% AcOH; (B) MeOH + 0.1% AcOH; gradient conditions: 0–15 min 80% (A)/20% (B), 15–25 min 10% (A)/90% (B); 25–30 min 80% (A)/20% (B). Injection volume: 20 μL. Reaction samples (150 μL) were diluted with MeOH (0.5 mL). Retention times (t_R_): ferulic acid = 5.6 min; (*E*)-(3-((3-(4-hydroxy-3-methoxyphenyl)acryloyl)oxy)propyl)triphenylphosphonium bromide (**SP2**): 6.5 min; 3-bromopropyl (*E*)-3-(4-hydroxy-3-methoxyphenyl) acrylate (**8**) = 11.7 min. All final products showed a purity of >95% by means of NMR spectroscopy.

### 2.1. Two-Step Flow Procedure for the Synthesis of MITO Compounds ***SP1-7***

A solution of phenolic acid (1 eq) in Cyrene (0.04–0.2 M, 2 mL) and a solution of 1,3-dibromopropane (2 eq) and DIPEA (2 eq) in Cyrene (2 mL) were mixed in a T-piece before entering a 10 mL reactor coil. Cyrene was used as flow stream. The temperature was set at 150 °C and the residence time at 30 min. The exiting flow stream containing the 3-bromopropyl ester was mixed in a T-piece with a solution of triphenylphosphine (2 eq) in Cyrene (4 mL) before entering a 10 mL reactor coil. The temperature was set at 200 °C and the residence time at 15 min. A 3-bar backpressure regulator was applied to the system. The flow stream was collected in a cooled flask containing toluene (20 mL) and a precipitate was formed. The supernatant was decanted, and the residue was washed with toluene and dried under reduced pressure. If necessary, the crude was purified by flash chromatography.

(*E*)-(3-((3-(4-hydroxyphenyl)acryloyl)oxy)propyl)triphenylphosphonium bromide (**SP1**). Starting solution: 0.04 M; Rf = 0.27 (dichloromethane/methanol 9:1); yield = 37%; light brown powder; m.p.: dec > 320 °C. ^1^H NMR (300 MHz, CD_3_OD) δ 7.94−7.71 (m, 15 H), 7.63 (d, *J* = 15.9 Hz, 1 H), 7.46 (d, *J* = 8.6 Hz, 2 H), 6.80 (d, *J* = 8.6 Hz, 2 H), 6.34 (d, *J* = 15.9 Hz, 1 H), 4.31 (t, *J* = 5.9 Hz, 2 H), 3.61−3.49 (m, 2 H), 2.15−2.01 (m, 2 H). ^13^C NMR (75 MHz, CD_3_OD) δ 167.5, 160.1, 145.6, 135.0 (d, *J* = 3.5), 133.4 (d, *J* = 10.4), 130.2 (d, *J* = 12.6), 129.8, 125.6, 118.2 (d, *J* = 87.0), 115.5, 113.2, 63.0 (d, *J* = 17.2), 21.9 (d, *J* = 3.5), 18.6 (d, *J* = 52.7).

(*E*)-(3-((3-(4-hydroxy-3-methoxyphenyl)acryloyl)oxy)propyl)triphenylphosphonium bromide (**SP2**). Starting solution: 0.2 M; Rf = 0.35 (dichloromethane/methanol 9:1); yield = 29%; yellow powder; m.p.: dec > 253 °C. ^1^H NMR (300 MHz, CD_3_OD) δ 7.94−7.72 (m, 15 H), 7.64 (d, *J* = 15.9 Hz, 1 H), 7.19 (d, *J* = 1.9 Hz, 1 H), 7.08 (dd, *J* = 8.2, 1.9 Hz, 1 H), 6.81 (d, *J* = 8.2 Hz, 1 H), 6.38 (d, *J* = 15.9 Hz, 1 H), 4.33 (t, *J* = 5.9 Hz, 2 H), 3.89 (s, 3 H), 3.63−3.52 (m, 2 H), 2.16−2.01 (m, 2 H). ^13^C NMR (75 MHz, CD_3_OD) δ 167.4, 149.5, 148.0, 145.9, 135.0 (d, *J* = 2.3), 133.4 (d, *J* = 9.2), 130.2 (d, *J* = 12.6), 126.1, 122.8, 118.2 (d, *J* = 85.9), 115.1, 113.5, 110.3, 63.0 (d, *J* = 18.4), 55.1, 22.0 (d, *J* = 3.4), 18.7 (d, *J* = 52.7).

(*E*)-(3-((3-(3,4-dihydroxyphenyl)acryloyl)oxy)propyl)triphenylphosphonium bromide (**SP3**). Starting solution: 0.1 M; Rf = 0.25 (dichloromethane/methanol 9:1); yield = 49%; yellow powder; m.p.: dec > 308 °C ^1^H NMR (300 MHz, CD_3_OD) δ 7.94−7.71 (m, 15 H), 7.57 (d, *J* = 15.9 Hz, 1 H), 7.04 (d, *J* = 2.1 Hz, 1 H), 6.95 (dd, *J* = 8.2, 2.1 Hz, 1 H), 6.78 (d, *J* = 8.2 Hz, 1 H), 6.27 (d, *J* = 15.9 Hz, 1 H), 4.32 (t, *J* = 5.7 Hz, 2 H), 3.62−3.50 (m, 2 H), 2.14−2.02 (m, 2 H). ^13^C NMR (75 MHz, CD3OD) δ 167.5, 148.4, 146.0, 145.5, 135.0 (d, *J* = 3.4), 133.4 (d, *J* = 10.3), 130.2 (d, *J* = 12.6), 126.2, 121.6, 118.2 (d, *J* = 85.6), 115.1, 113.8, 113.2, 63.0 (d, *J* = 18.3), 21.9 (d, *J* = 3.4), 18.6 (d, *J* = 52.7).

(*E*)-(3-((3-(4-hydroxy-3,5-dimethoxyphenyl)acryloyl)oxy)propyl)triphenylphosphonium bromide (**SP4**). Starting solution: 0.08 M; Rf = 0.33 (dichloromethane/methanol 9:1); yield = 40%; yellow powder; m.p.: dec > 282 °C. ^1^H NMR (300 MHz, CD_3_OD) δ 7.94−7.73 (m, 15 H), 7.64 (d, *J* = 15.9 Hz, 1 H), 6.92 (s, 2 H), 6.42 (d, *J* = 15.9 Hz, 1 H), 4.33 (t, *J* = 5.8 Hz, 2 H), 3.88 (s, 6 H), 3.64−3.52 (m, 2 H), 2.16−2.02 (m, 2 H). ^13^C NMR (75 MHz, CD_3_OD) δ 167.3, 148.1, 146.1, 138.5, 135.0 (d, *J* = 2.3), 133.4 (d, *J* = 10.3), 130.2 (d, *J* = 12.6), 125.1, 118.2 (d, *J* = 87.0), 114.0, 105.6, 63.0 (d, *J* = 18.3), 55.5, 21.9 (d, *J* = 3.4), 18.6 (d, *J* = 53.8).

(3-((4-hydroxy-3,5-dimethoxybenzoyl)oxy)propyl)triphenylphosphonium bromide (**SP5**). Starting solution: 0.1 M; Rf = 0.44 (dichloromethane/methanol 9:1) Yield = 50%; white powder m.p.: dec > 230 °C; ^1^H NMR (300 MHz, CD_3_OD) δ 7.93−7.71 (m, 15 H), 7.32 (s, 2 H), 4.44 (t, *J* = 6.0 Hz, 2 H), 3.87 (s, 6 H), 3.66−3.55 (m, 2 H), 2.22−2.11 (m, 2 H). ^13^C NMR (75 MHz, CD_3_OD) δ 166.3, 147.6, 141.1, 135.0 (d, *J* = 3.4), 133.4 (d, *J* = 9.2), 130.2 (d, *J* = 12.6), 119.4, 118.2 (d, *J* = 87.0), 107.0, 63.6 (d, *J* = 18.4), 55.5, 22.0, 18.6 (d, *J* = 53.8).

Triphenyl(3-((3,4,5-trihydroxybenzoyl)oxy)propyl)phosphonium bromide (**SP6**). Starting solution: 0.20 M; Rf = 0.27 (dichloromethane/methanol 9:1); yield = 21%; yellow powder; m.p.: dec > 282 °C; ^1^H NMR (300 MHz, CD_3_OD) δ 7.94−7.71 (m, 15 H), 7.05 (s, 2 H), 4.38 (t, *J* = 5.9 Hz, 2 H), 3.62−3.48 (m, 2 H), 2.18−2.04 (m, 2 H). ^13^C NMR (75 MHz, CD_3_OD) δ 166.6, 158.0, 145.2, 135.0 (d, *J* = 2.0), 133.4, 133.4 (d, *J* = 10.2), 130.2 (d, *J* = 12.6), 118.1 (d, *J* = 87.0), 108.8, 63.2 (d, *J* = 18.3), 22.0, 18.6 (d, *J* = 53.8).

(*R,E*)-(3-((3-(3,4-dihydroxyphenyl)-2-((3-(3,4-dihydroxyphenyl)acryloyl)oxy)propanoyl)oxy)propyl)triphenylphosphonium bromide (**SP7**). Starting solution: 0.20 M; Rf = 0.23 (dichloromethane/methanol 9:1); yield = 18%; yellow powder; m.p.: dec > 265 °C ^1^H NMR (300 MHz, CD_3_OD) δ 7.90−7.67 (m, 15 H), 7.46 (d, *J* = 15.9 Hz, 1 H), 6.94 (d, *J* = 2.0 Hz, 1 H), 6.83 (dd, *J* = 8.3, 2.0 Hz, 1 H), 6.74 (d, *J* = 8.3 Hz, 1 H), 6.72 (d, *J* = 2.0 Hz, 1 H), 6.63 (d, *J* = 8.0 Hz, 1 H), 6.56 (dd, *J* = 8.0, 2.0 Hz, 1 H), 6.23 (d, *J* = 15.9 Hz, 1 H), 5.16 (t, *J* = 6.4 Hz, 1 H), 4.29−4.14 (m, 2 H), 3.28−3.21 (m, 2 H), 3.08−3.04 (m, 2 H), 1.97−1.87 (m, 2 H) [25]. ^13^C NMR (75 MHz, CD_3_OD) δ 170.2, 167.3, 148.9, 146.9, 145.6, 144.8, 144.1, 135.0, 133.3 (d, *J* = 9.8), 130.2 (d, *J* = 12.6), 127.0, 125.7, 121.9, 120.4, 118.2 (d, *J* = 86.2), 116.2, 115.0, 114.9, 113.8, 112.3, 73.7, 63.5 (d, *J* = 18.3), 36.3, 29.2, 21.7, 18.6 (d, *J* = 53.8).

### 2.2. Cell Cultures and Treatment

Neuronal cell line SH-SH5Y at a density of 15,000 cells/well on 96-well plates was cultured with DMEM F12 and supplemented with 10% fetal bovine serum (FBS), 100 U/mL penicillin and 100 U/mL streptomycin (Sigma) and 2 mM L-glutamine in a humidified atmosphere of 95% air and 5% CO_2_ at 37 °C. For IC_50_, the cells were treated with the MITO compounds at the following concentrations: 3.5, 7.5, 16.6, 31.5, 62.5, 125, 250, 500 µM and 1 mM. For acute ROS generation, 500 µM of *tert*-butyl hydroperoxide (Luperox^®^ TBH70X, Merck Life Science S.r.l., Milan, Italy) was used for 2 h, alone and in combination with the MITO compounds, for cell viability assay and morphology analysis. During pretreatment experiments, the MITO compounds were removed before the TBH stimulation. The control (CTRL) groups received an equal volume of the medium.

### 2.3. Cell Viability and Morphology

Cells were grown at a density of 15,000 cells/well on 96-well plates in a final volume of 100 µL/well. After the treatments, cell viability was assessed by MTS assay (Promega Italia S.r.l., Milan, Italy), by absorbance measurements at a wavelength of 490 nm, after 2 h incubation at 37 °C. Cell viability was expressed by normalization with the control. A Zeiss Axio Scope 2 microscope (Carl Zeiss, Oberkochen, Germany) was used for the analysis of cell morphology.

### 2.4. Cell Morphology

Cells were grown at a density of 15,000 cells/well on glass coverslips 12 mm in diameter. At the end of treatments, cells were fixed with 4% formaldehyde solution for 15 min at room temperature, washed twice with PBS 1x, and the coverslips were mounted on slides. Twelve bright-field images (20×)/coverslip were obtained using a Leica DMIL LED inverted microscope equipped with a Leica camera. The cell’s body was measured by manually tracing the surface of the cell using ImageJ 1.52v software (National Institutes of Health, Bethesda, MD, USA). All the counts were carried out in a blind manner by two independent experimenters unaware of sample identity. The average value of the cellular body was employed for statistical analysis.

### 2.5. Statistical Analysis

Data analysis was performed using GraphPad Prism 8.4.3 software (GraphPad Software, Inc, La Jolla, CA, USA). All samples were tested in triplicate and values reported were obtained as the mean of at least three independent experiments ± standard error (SE). Statistical evaluations were performed using one-way ANOVA followed by Tukey’s post hoc test. Differences in *p*-value less than 0.05 were considered statistically significant.

## 3. Results and Discussion

### 3.1. Continuous Flow Synthesis of MITO Compounds ***SP1-7***

**Scheme 1:**
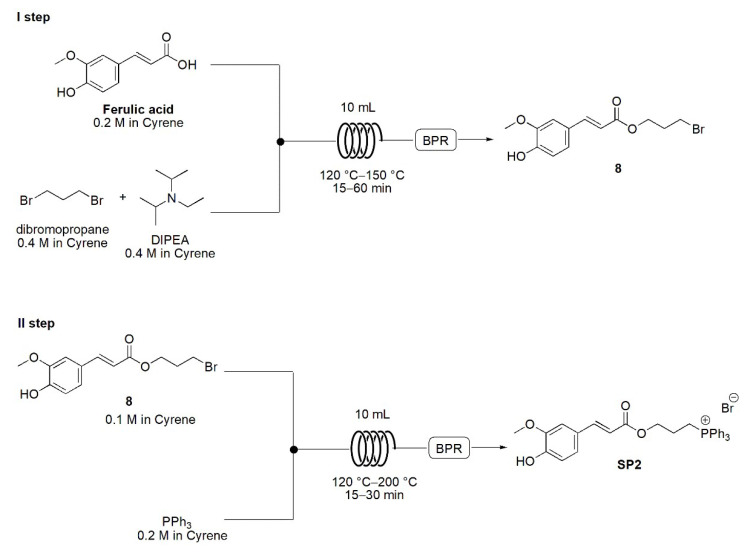
Flow synthesis of compound **SP2** in Cyrene.

First attempts to synthesize the target compounds were performed in batch using ferulic acid as model phenolic starting material and (3-hydroxypropyl) triphenylphosphonium bromide, previously synthesized following a literature procedure [26]. Different conditions, catalysts and coupling reagents were screened [25,27,28,29], but the reaction proceeded very slowly, leaving most of the starting material unreacted and forming complex mixtures in which it was not possible to identify the desired product. Therefore, the chemical esterification was tried using 3-bromopropan-1-ol and different coupling reagents (DCC/DMAP, HOBT/EDC), but again, complex mixtures were formed and the desired ester was isolated in very low yields (5–7%). Finally, a different approach was exploited, using 1,3-dibromopropane in the presence of a base in acetonitrile at reflux for 2 days to form the corresponding bromopropyl ferulic ester in 29% yield [24]. The intermediate was then reacted with Ph_3_P at reflux in toluene for 6 days to yield the final product **SP2** (24% yield). Overall, the two-step batch procedure produced the desired MITO derivative with a yield of about 7% after prolonged reaction time (8 days in total) under heating. Therefore, we decided to develop a more versatile and convenient synthesis under flow conditions. Due to the different solubility of the starting phenolic acids, acetonitrile was not a suitable solvent for all the starting materials. To avoid the use of DMF or DMSO, Cyrene was selected as the solvent. First, the two steps were studied separately. A solution of ferulic acid in Cyrene (0.2 M) and a solution of 1,3-dibromopropane (2 eq) and DIPEA (2 eq) in Cyrene were mixed in a T-piece and reacted in a reactor coil (10 mL) (Scheme 1, I step). Temperature and residence time were varied (temperature: 120 °C, 135 °C and 150 °C; residence time: 15, 30 and 60 min) and the reaction outcome was monitored by HPLC. At 150 °C, in 30 min of residence time, a conversion of 90% was achieved.

**Scheme 2:**
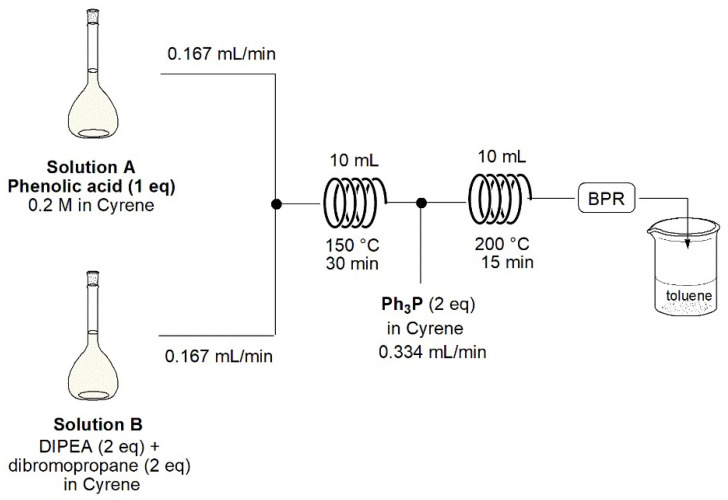
Optimized flow reactor configuration for the synthesis of the MITO compounds.

Then, the crude solution obtained from step I was mixed with a solution of triphenylphosphine (2 eq) in Cyrene and reacted in a reactor coil (10 mL) (Scheme 1, II step). Different temperatures and residence times were tested, and the reaction outcome was monitored by HPLC. Working at 120 °C, no conversion was obtained, whereas at 150 °C only 35% of conversion was achieved in 30 min. In 15 min at 200 °C, a conversion of 67% was reached; by increasing the residence time (30 min), a similar conversion was obtained (70%). Finally, a two-step automated protocol was developed (Scheme 2) by mixing the flow stream containing compound **8** with a third flow stream of Ph_3_P in Cyrene; the resulting mixture was directly reacted into a 10 mL reactor coil. The exiting solution was collected into a cooled flask containing toluene that led to the precipitation of the crude product, which was then decanted, dried, and purified by flash chromatography. Exploiting this strategy, all the MITO compounds **SP1-7** were obtained in only 45 min of residence time, in moderate to good isolated yields (Table S1). The lower yields were obtained in the synthesis of gallic acid and rosmarinic acid derivatives (21% and 18%, respectively). In both cases, incomplete conversion was observed by HPLC analyses; however, attempts to increase conversion by increasing temperature and residence time were not successful due to the formation of complex reaction mixtures, difficult to purify.

### 3.2. Effects of the MITO Compounds ***SP1-7*** on Neuronal Cell Viability

**Figure 2:**
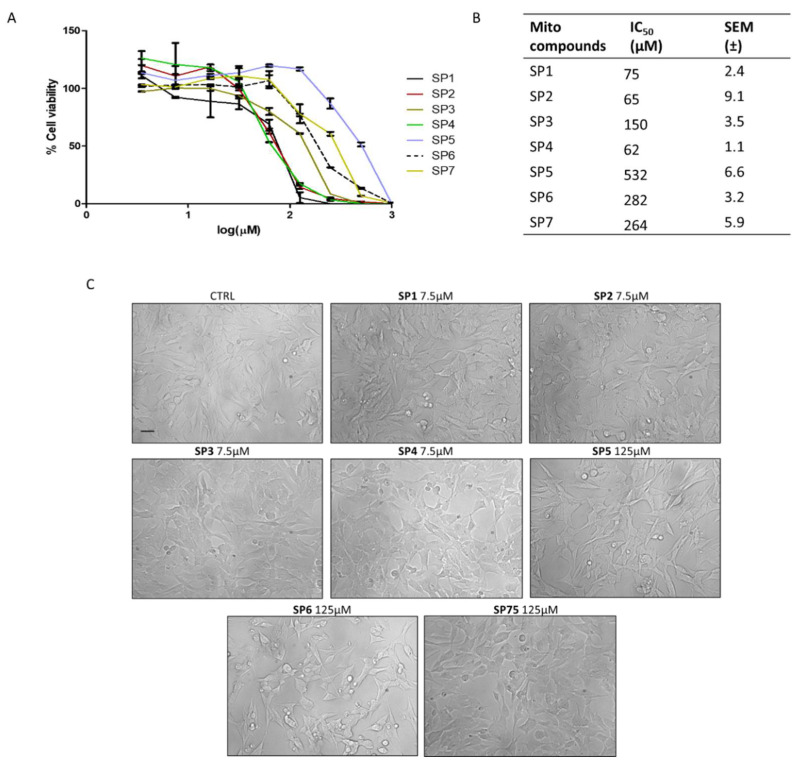
Effects of the MITO compounds **SP1-7** on cell viability and morphology of SH-SY5Y cells for 24 h. (**A**) MTS cell viability assay at 3.5, 7.5, 16.6, 31.5, 62.5, 125, 250, 500 µM and 1 mM for 24 h. (**B**) IC_50_ concentrations calculated in GraphPad Prisma 8.4.3 software. Fraction of live cells (%) is provided on vertical axis and log concentration (µM) on horizontal axis. The IC_50_ is the concentration at which the curve passes through the 50% inhibition level. (**C**) Representative morphological images of untreated cells (CTRL) and cells treated with the MITO compounds. Bar: 50 μm.

To determine cytocompatibility, we performed a dose–effect analysis on cell viability to MITO compounds **SP1-7** on SH-SY5Y neuronal cells. To this aim, we evaluated large-range doses for 24 and 72 h to determine the IC_50_. The diagram in Figure 2A shows the viability effect of the MITO compounds in a concentration-dependent manner after the 24 h of treatment. The IC_50_ values were calculated and are shown in Figure 2B. The results evidenced that the maximum concentration of the MITO compounds that did not induce any significant change in cell viability during the 24 h assay was 7.5 µM for **SP1**, **SP2**, **SP3**, and **SP4** and 125 µM for **SP5**, **SP6** and **SP7** (Figure 2C). These results were confirmed by morphological analyses (Figure 2C). Indeed, in all treatments, the neuronal cells presented a normal cellular body (from 40 to 70 μM) and numerous dendrites were present. This indicated the normal cell morphology of cultured neurons. Furthermore, in order to evaluate the effect of the long stimulation, the IC_50_ was measured after 72 h of MITO compound stimulation, and the data are show in the supplementary (Figure S1).

### 3.3. Biological Effects of the MITO Compounds **SP1-7** on Neuronal Cells

**Figure 3:**
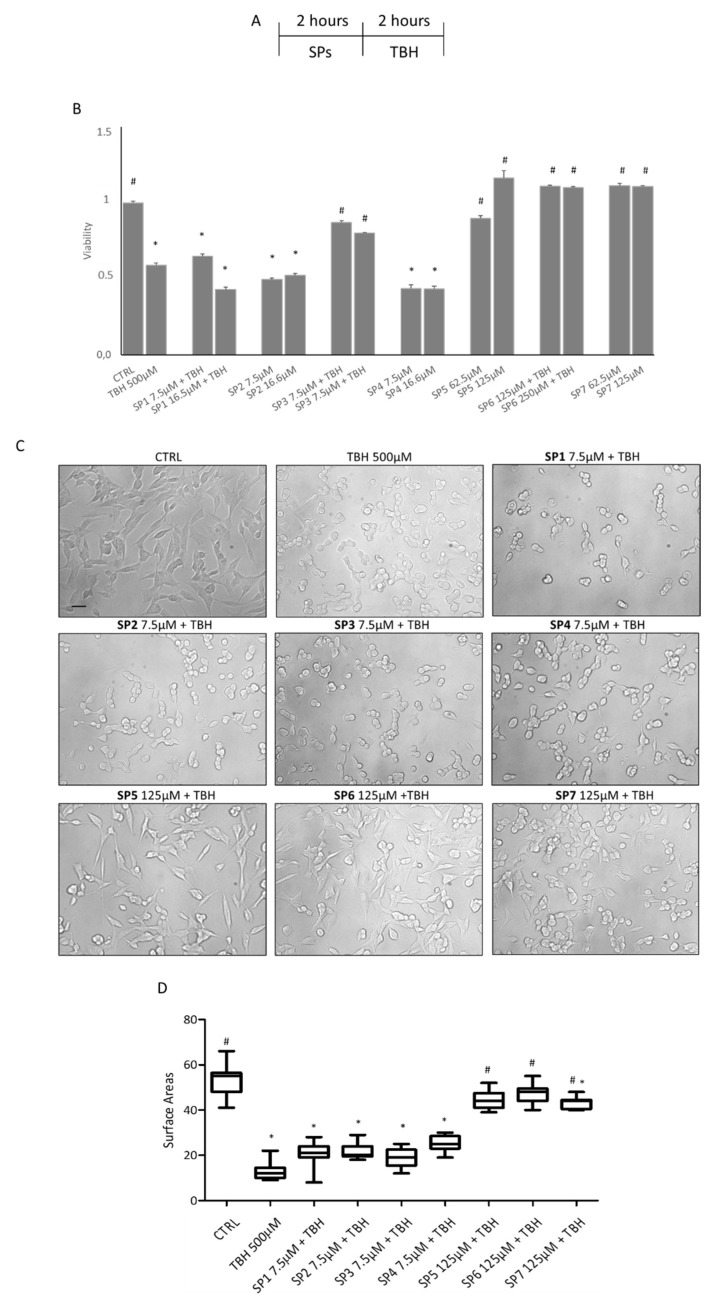
Antioxidant and protective effects of MITO compound treatment against TBH-induced oxidative stress, pretreatment. (**A**) Schematic representation of the pretreatment with the MITO compounds. (**B**) MTS assay of cytoprotective effect of the MITO compounds (2 h) on cells pretreated with TBH (2 h, 500 µM). (**C**) Representative morphological images. (**D**) Graphic of cellular body areas. Bar: 50 μm. Tukey test: * *p* < 0.05 compared to CTRL group; # *p* < 0.01 compared to TBH group.

To evaluate the effects of the MITO compounds on acute oxidative stress, we analyzed their biological activity using a high concentration of the oxidative agent TBH to induce oxidative stress on neuronal cells. Neuronal cells are able to counteract small variations in the level of ROS, but an intense level of oxidative stress is uncompensated from the endogenous antioxidant system. The ability to protect cellular mitochondria from oxidative insult was analyzed by MTS assay, which is based on the mitochondrial respiration/activity and serves to indirectly evaluate cellular energy capacity. In the pretreatment experiments, shown in Figure 3A, SH-SY5Y cells were treated with the MITO compounds for 2 h; then, they were removed and 500 µM TBH was added for 2 h to induce oxidative stress. Treatment with TBH induced a 40% reduction in cell viability, as reported in Figure 3B. Despite the ability of **SP3**, **SP5**, **SP6** and **SP7** to protect cell viability from oxidative insult, only **SP5** and **SP6** maintained morphological integrity (Figure 3C,D). In fact, dimensions of the cellular body and neurite formation were similar to the control.

**Figure 4:**
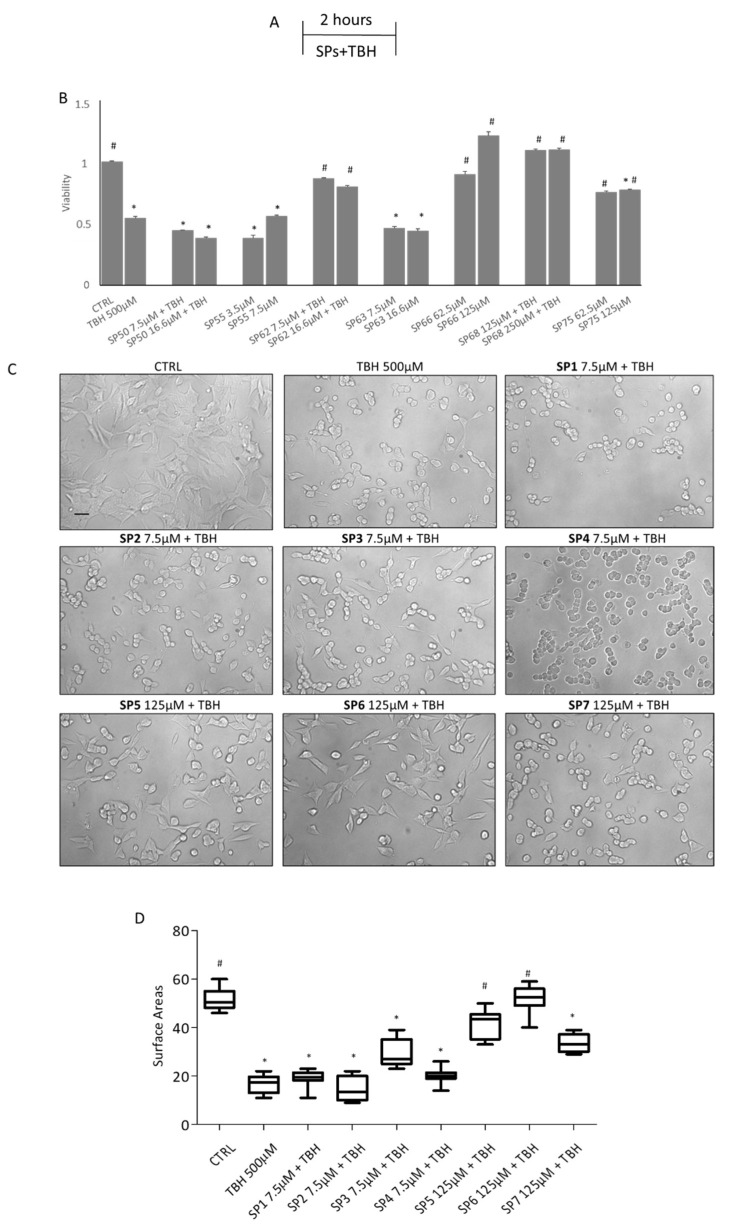
Antioxidant and protective effects of MITO compound treatment against TBH-induced oxidative stress. (**A**) Schematic representation of cotreatment with the MITO compounds. (**B**) MTS assay of cytoprotective effect of the MITO compounds cotreated with TBH (2 h, 500 µM). (**C**) Representative morphological images. (**D**) Graphic of cellular body areas. Bar: 50 μm. Tukey test: * *p* < 0.05 compared to CTRL group; # *p* < 0.01 compared to TBH group.

A cotreatment experiment was designed, as shown in Figure 4A. SH-SY5Y cells were treated with the MITO compounds in combination with TBH at 500 µM for 2 h (Figure 4A). The cell viability was similar to the control after **SP3, SP5**, **SP6 and SP7** cotreatment with THB (Figure 4B). Moreover, they were able to protect the cellular morphology from oxidative stress (Figure 4C,D). In contrast, **SP3** and **SP7** induced the recovery of cellular viability, but the value did not reach the control; this could explain the absence of recovery of cellular morphology.

## 4. Conclusions

MITO compounds represent an exciting opportunity to protect the mitochondria from excessive ROS production that can damage mitochondria and lead to neurodegeneration. However, biological considerations concerning MITO compound safety should be evaluated. For this aim, we studied the cytocompatibility of seven synthetized MITO compounds using human neuronal cell line SH-SH5Y for the evaluation of safety and mitoprotection effects. The safe dose depends on the type of compound synthesized, and the non-toxic dose is between 3.5 and 125 µM. The MITO compounds that have interesting performances are **SP5** and **SP6**, both of which have the ability to protect mitochondria from oxidative insult during pre- and cotreatment. Further structure–activity relationship studies will be performed, modifying the length and consequently the hydrophobicity of the alkyl chain spacer, in order to evaluate and fine-tune their membrane binding and distribution within mitochondria and cells.

**Figure 5:**
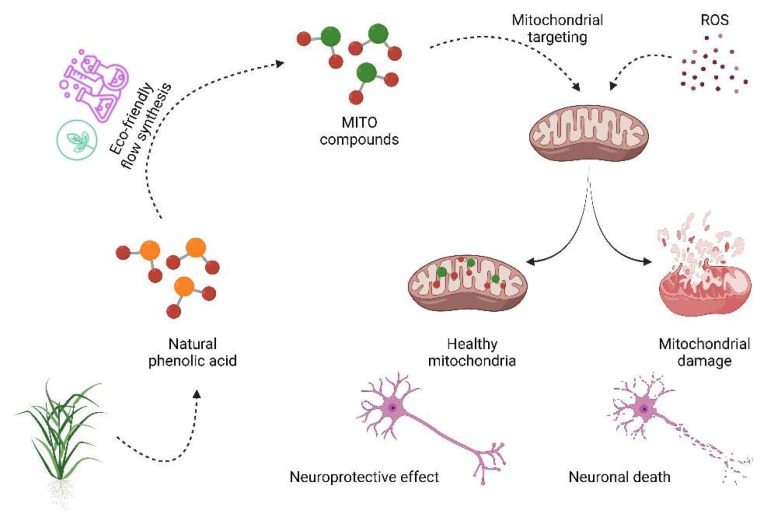
Schematic representation of the effects of the MITO compounds. Created with BioRender.com.

This first study made it possible to select the most promising MITO compounds, and lays the foundations for planning future studies, necessary to evaluate and confirm through biochemical assays the mitoprotective effect of the selected compounds and to study the different mitochondrial activities and functions (Figure 5).

## Data Availability

Data are contained within the article and supplementary materials.

## Supplementary Materials

The following supporting information can be downloaded at: https://www.mdpi.com/article/10.3390/antiox11112160/s1, Table S1: Overall yields obtained exploiting the two-step flow procedure reported in Paragraph 2.1; Figure S1: Effects of the MITO compounds **SP1-7** on cell viability and morphology of SH-SY5Y cells for 72 hours; NMR spectra of compounds **SP1-7**.
