# Supplementary material for: Flow Synthesis of Nature-Inspired Mitochondria-Targeted Phenolic Derivatives as Potential Neuroprotective Agents
Source: Antioxidants (Basel). 2022 Oct 31;11(11):2160. doi: 10.3390/antiox11112160 (PMC9686576; doi:10.3390/antiox11112160)

Supporting information

Flow synthesis of nature-inspired mitochondria-targeted phenolic derivatives as potential neuroprotective agents

Desirée Pecora,[1] Francesca Annunziata,[1] Sergio Pegurri,[1] Pasquale Picone,[2] Andrea Pinto,[3] Domenico Nuzzo,[2,*] Lucia Tamborini[1,*]

[1]Department of Pharmaceutical Sciences, University of Milan, via Mangiagalli 25, 20133 Milan, Italy.

[2]Istituto per la Ricerca e l'Innovazione Biomedica (IRIB), CNR, Via Ugo La Malfa 153, 90146 Palermo, Italy.

[3]Department of Food, Environmental and Nutritional Sciences, University of Milan, via Celoria 2, 20133 Milan, Italy.

*Correspondence: lucia.tamborini@unimi.it; Tel.: + 39 0250319367; domenico.nuzzo@irib.cnr.it; Tel: +39 0916809518

Contents

- Table S1 page 2
- Figure S1 page 3
- NMR spectra page 4

Table S1. Overall yields obtained exploiting the two-step flow procedure reported in Paragraph 2.1

Compound	Yield (%)
**SP1**	37
**SP2**	29
**SP3**	49
**SP4**	40
**SP5**	50
**SP6**	21
**SP7**	18

A

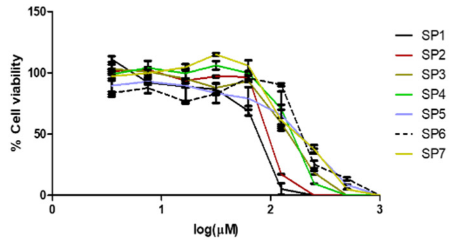

B

Mito compounds	IC_{50} (μM)	SEM (±)
SP1	75	3,3
SP2	102	5,1
SP3	149	2,6
SP4	150	2,2
SP5	521	3,7
SP6	237	1,9
SP7	173	3,5

C

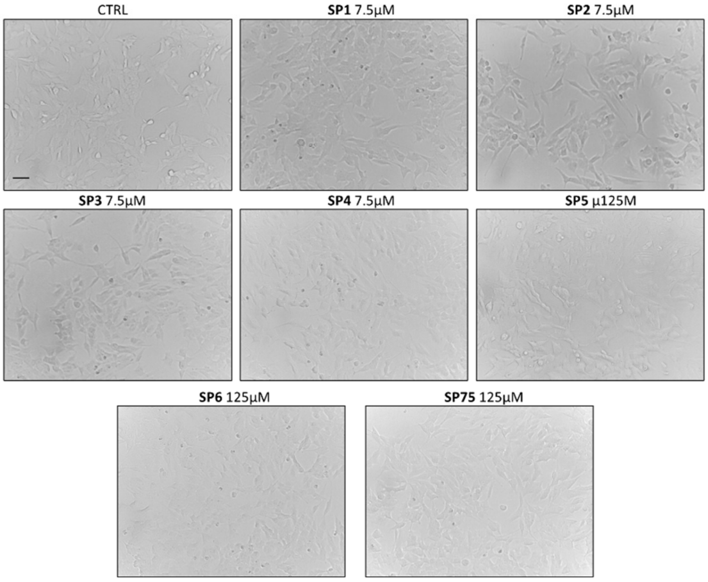

Figure S1. Effects of the MITO compounds **SP1-7** on cell viability and morphology of SH-SY5Y cells for 72 hours. A) MTS cell viability assay at 3.5, 7.5, 16.6, 31.5, 62.5,125, 250, 500 μM and 1 mM for 72 hours. B) IC_{50} concentrations calculated in GraphPad Prisma 8.4.3 software, fraction of alive cells (%) is provided on vertical axis and log concentration (μM) on horizontal axis. The IC_{50} is the concentration at which the curve passes through the 50% inhibition level. C) Representative morphological images of untreated cells (CTRL) and cells treated with the MITO compounds.

^{1}H and ^{13}C NMR spectra of compounds SP1-7

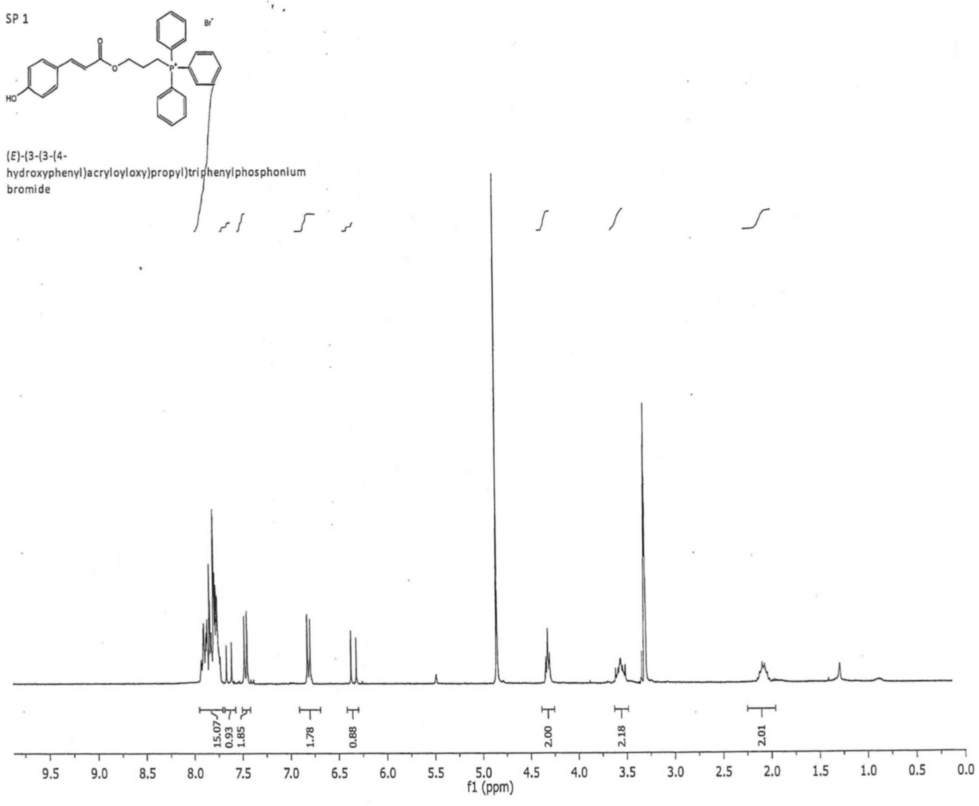

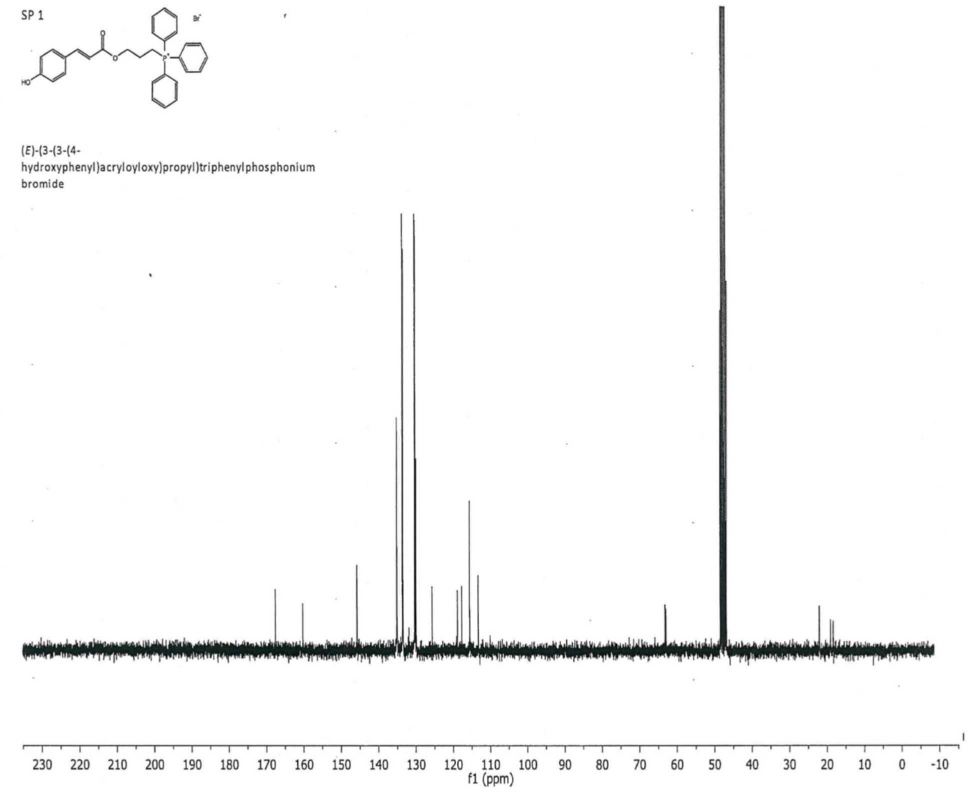

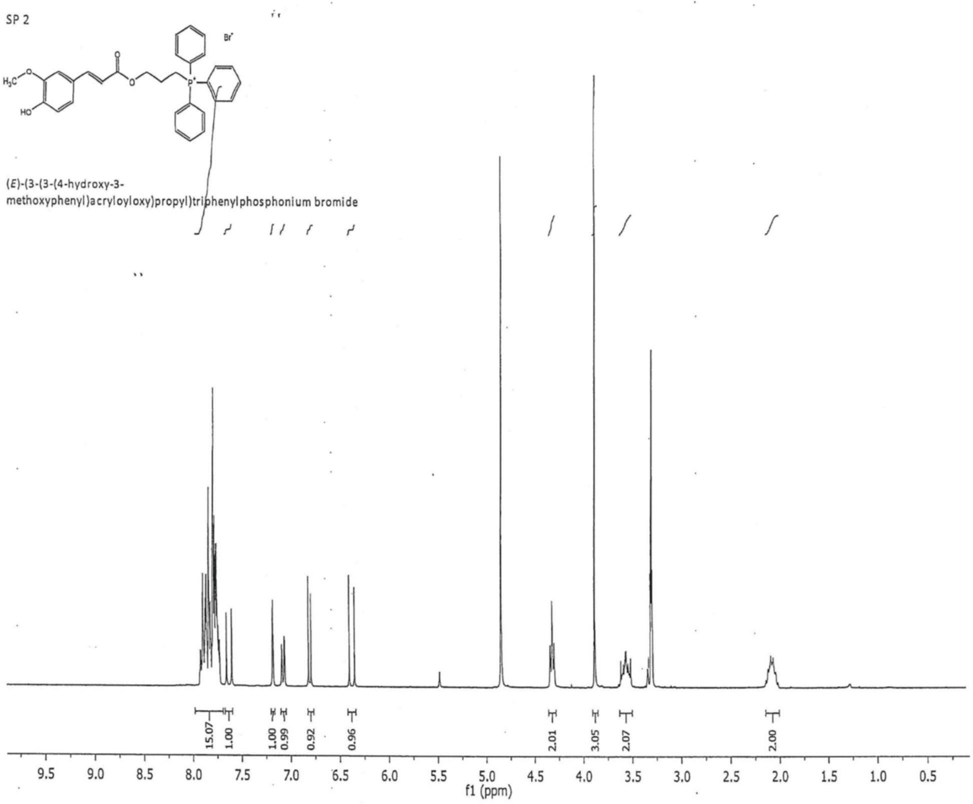
SP 2
Br⁻
H3C
HO
(E)-(3-(3-(4-hydroxy-3-methoxyphenyl)acryloyloxy)propyl)triphenylphosphonium bromide
15.07
1.00
1.00
0.99
0.92
0.96
2.01
3.05
2.07
2.00
9.5 9.0 8.5 8.0 7.5 7.0 6.5 6.0 5.5 5.0 4.5 4.0 3.5 3.0 2.5 2.0 1.5 1.0 0.5
f1 (ppm)

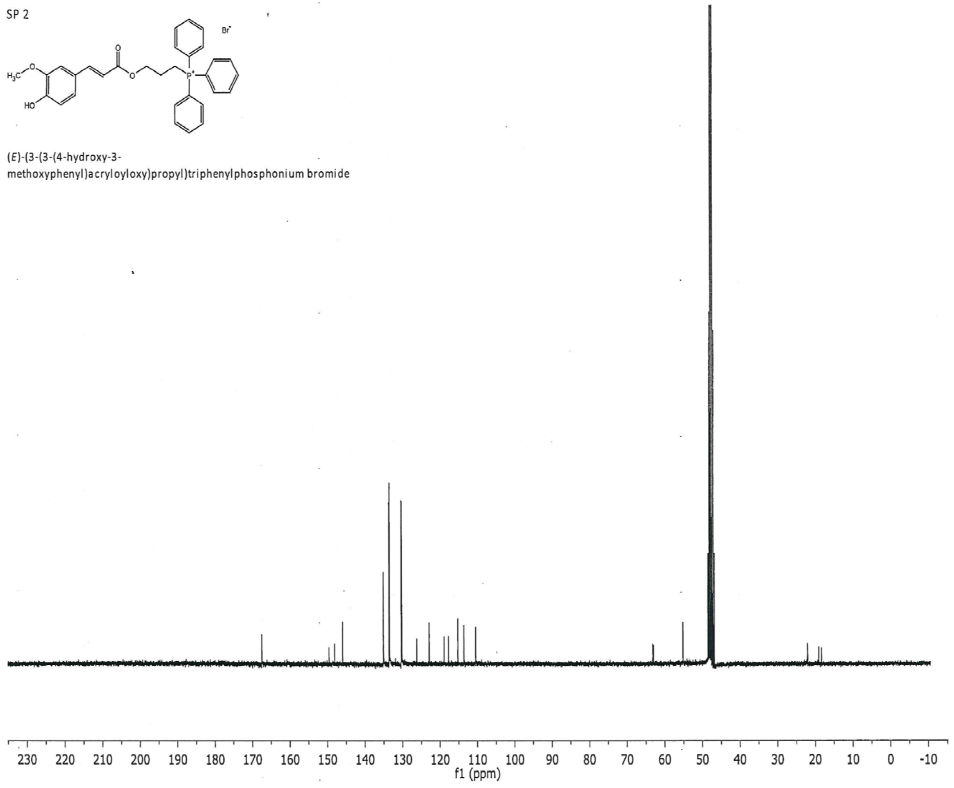
SP 2
Br⁻
H3C
HO
(E)-(3-(3-(4-hydroxy-3-methoxyphenyl)acryloyloxy)propyl)triphenylphosphonium bromide
230 220 210 200 190 180 170 160 150 140 130 120 110 100 90 80 70 60 50 40 30 20 10 0 -10
f1 (ppm)

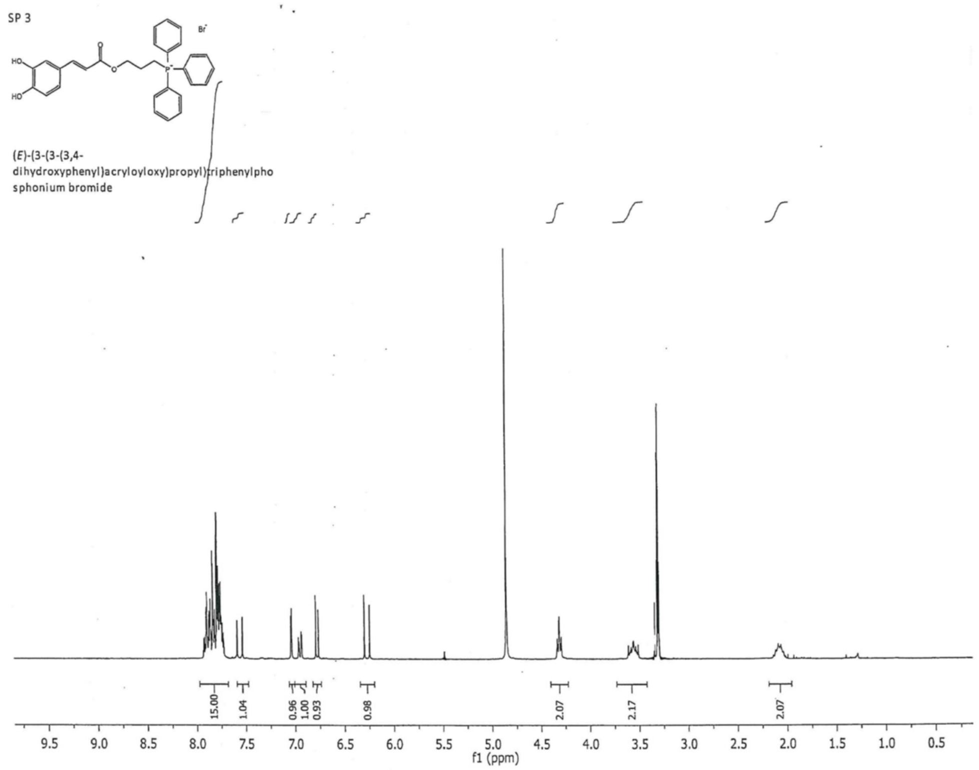
SP 3
Br⁻
HO
HO
(E)-(3-(3-(3,4-dihydroxyphenyl)acryloyloxy)propyl)triphenylphosphonium bromide
15.00
1.04
0.96
1.00
0.93
0.98
2.07
2.17
2.07
9.5 9.0 8.5 8.0 7.5 7.0 6.5 6.0 5.5 5.0 4.5 4.0 3.5 3.0 2.5 2.0 1.5 1.0 0.5
f1 (ppm)

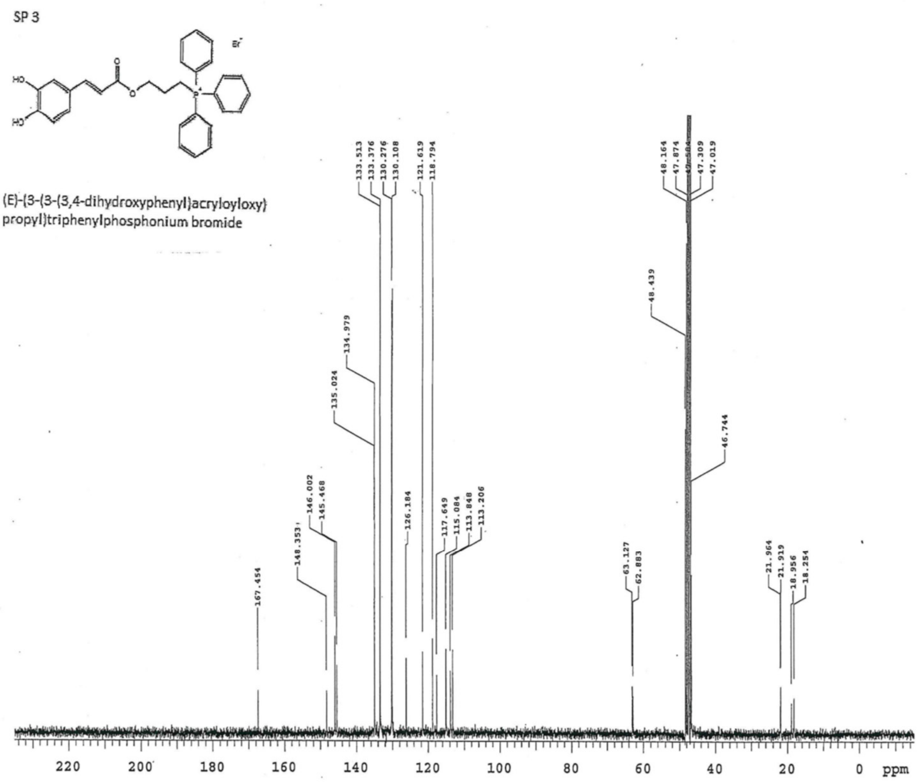
SP 3
Br⁻
HO
HO
(E)-(3-(3-(3,4-dihydroxyphenyl)acryloyloxy)
propyl)triphenylphosphonium bromide
167.454
148.353
146.002
145.468
135.024
134.979
133.513
133.376
130.276
130.108
126.184
121.619
118.794
117.649
115.084
113.848
113.206
63.127
62.883
48.439
48.164
47.874
47.309
47.019
46.744
21.964
21.919
18.956
18.254
220 200 180 160 140 120 100 80 60 40 20 0 ppm

SP 4

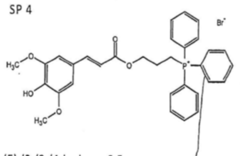

(E)-(3-(3-(4-hydroxy-3,5-dimethoxyphenyl)acryloyloxy)propyl)triphenylphosphonium bromide

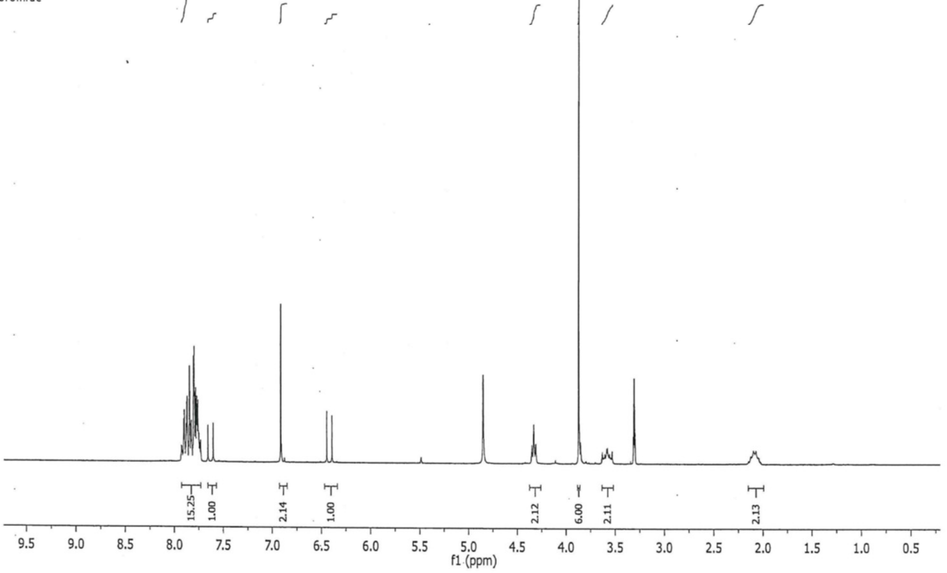

SP 4

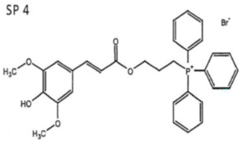

(*E*)-(3-(3-(4-hydroxy-3,5-dimethoxyphenyl)acryloyloxy)propyl)triphenylphosphonium bromide

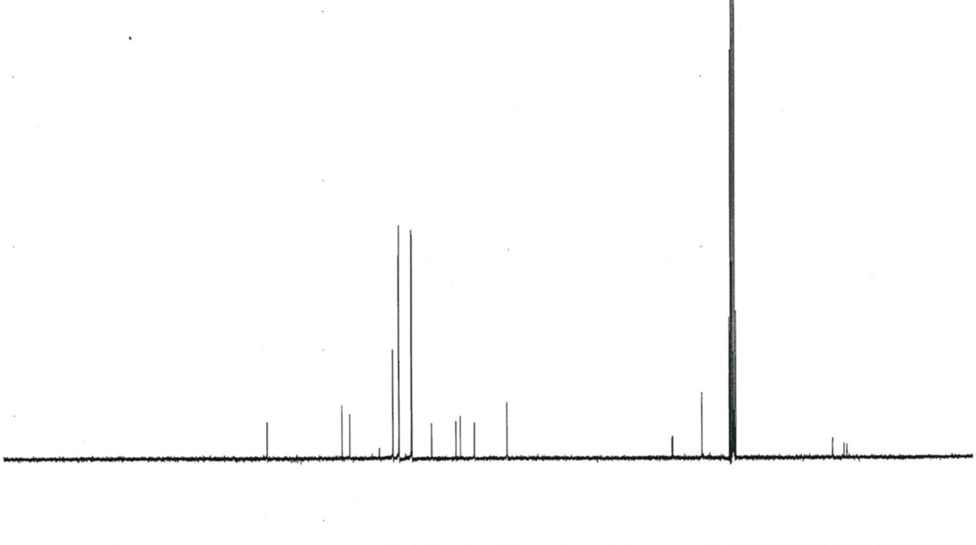

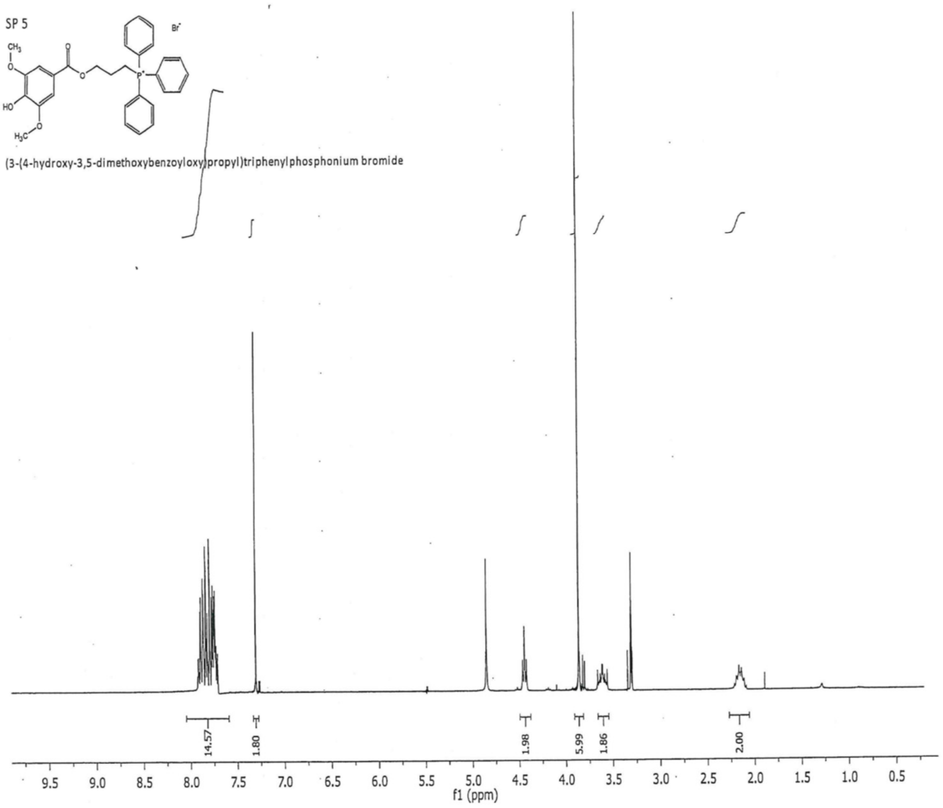
SP 5
(3-(4-hydroxy-3,5-dimethoxybenzoyloxy)propyl)triphenylphosphonium bromide
14.57
1.80
1.98
5.99
1.86
2.00
9.5 9.0 8.5 8.0 7.5 7.0 6.5 6.0 5.5 5.0 4.5 4.0 3.5 3.0 2.5 2.0 1.5 1.0 0.5
f1 (ppm)

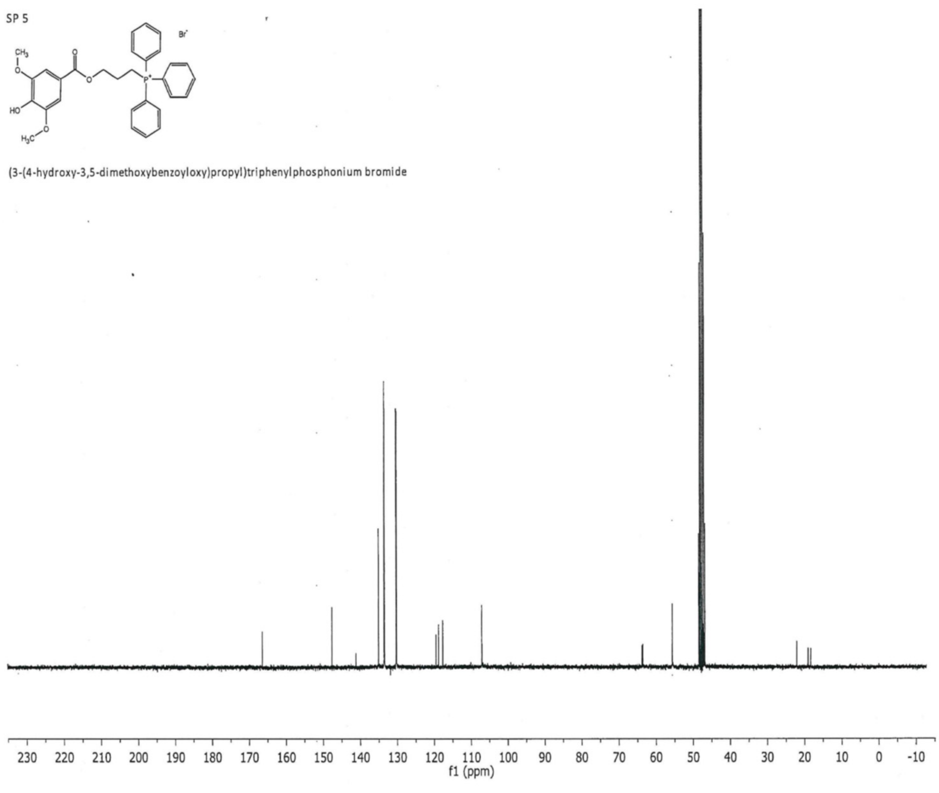
SP 5
(3-(4-hydroxy-3,5-dimethoxybenzoyloxy)propyl)triphenylphosphonium bromide
230 220 210 200 190 180 170 160 150 140 130 120 110 100 90 80 70 60 50 40 30 20 10 0 -10
f1 (ppm)

SP 6

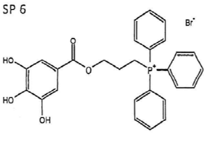

triphenyl(3-(3,4,5-trihydroxybenzoyloxy)propyl)phosphonium bromide

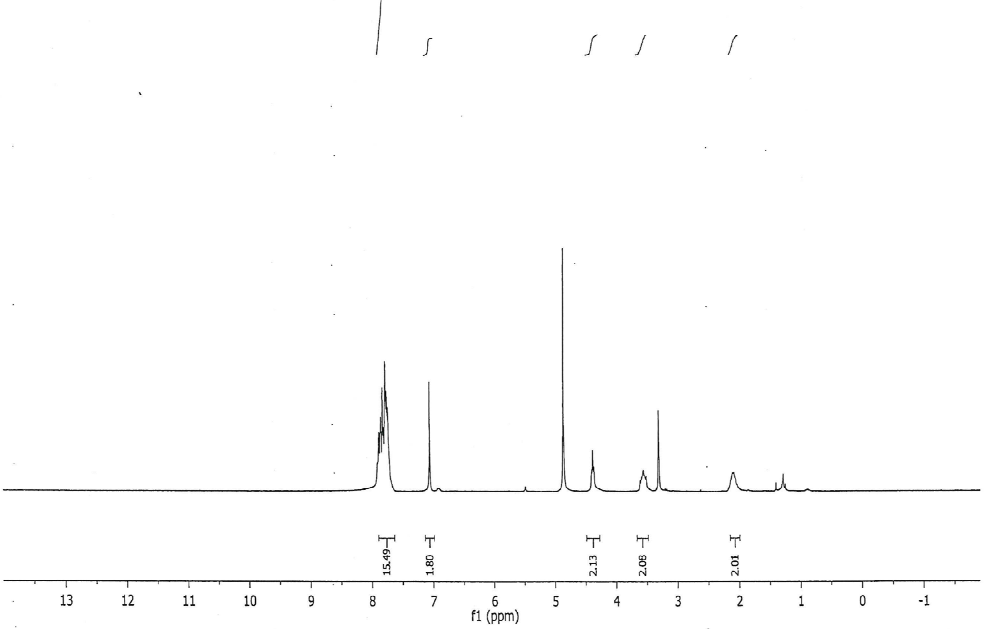

SP 6

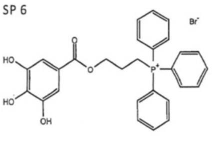

triphenyl(3-(3,4,5-trihydroxybenzoyloxy)propyl)phosphonium bromide

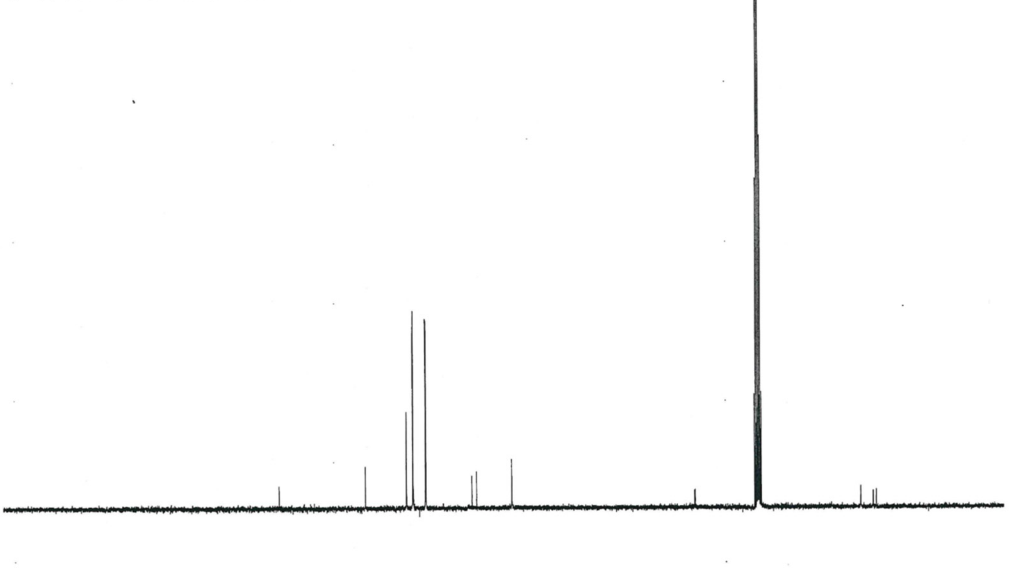

SP 7

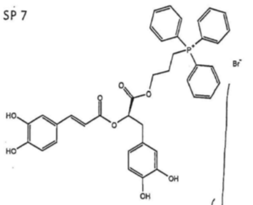

(*R*,*E*)-(3-(3-(3,4-dihydroxyphenyl)-2-(3-(3,4-dihydroxyphenyl)acryloyloxy)propanoyloxy)propyl) triphenylphosphonium bromide

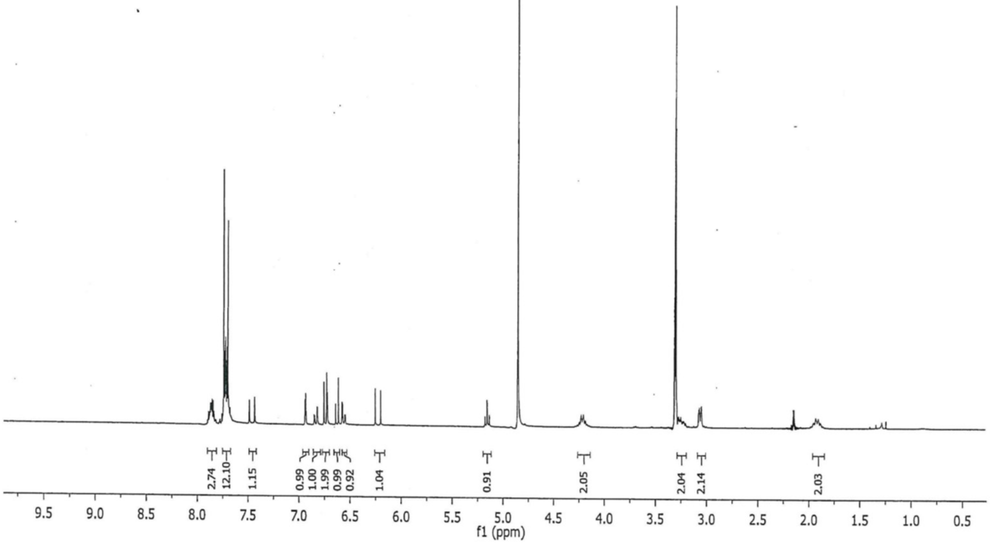

SP 7

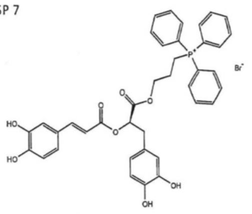

(*R*,*E*)-(3-(3-(3,4-dihydroxyphenyl)-2-(3-(3,4-dihydroxyphenyl)acryloyloxy)propanoyloxy)propyl) triphenylphosphonium bromide

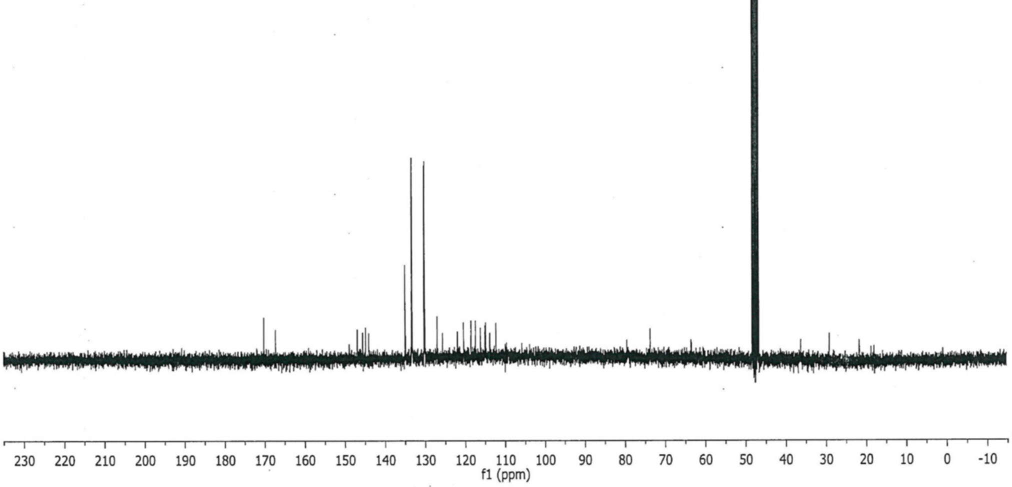

Supplement: Supplementary file 1 [file antioxidants-11-02160-s001.zip › antioxidants-1938831-supplementary.pdf]
